# Inhibitory Effect and Possible Mechanism of Action of Patchouli Alcohol against Influenza A (H2N2) Virus

**DOI:** 10.3390/molecules16086489

**Published:** 2011-08-03

**Authors:** Huaxing Wu, Beili Li, Xue Wang, Mingyuan Jin, Guonian Wang

**Affiliations:** 1 Department of Endoscopy, the Third Affiliated Hospital, Harbin Medical University, Harbin 150040, China; 2 Department of Anesthesiology, the Third Affiliated Hospital, Harbin Medical University, Harbin 150081, China

**Keywords:** patchouli alcohol, docking, neuraminidase, MTT, influenza virus, mice

## Abstract

In the present study, the anti-influenza A (H2N2) virus activity of patchouli alcohol was studied *in vitro*, *in vivo* and *in silico*. The CC_50_ of patchouli alcohol was above 20 µM. Patchouli alcohol could inhibit influenza virus with an IC_50_ of 4.03 ± 0.23 µM. MTT assay showed that the inhibition by patchouli alcohol appears strongly after penetration of the virus into the cell. In the influenza mouse model, patchouli alcohol showed obvious protection against the viral infection at a dose of 5 mg/kg/day. Flexible docking and molecular dynamic simulations indicated that patchouli alcohol was bound to the neuraminidase protein of influenza virus, with an interaction energy of –40.38 kcal mol^–1^. The invariant key active-site residues Asp151, Arg152, Glu119, Glu276 and Tyr406 played important roles during the binding process. Based on spatial and energetic criteria, patchouli alcohol interfered with the NA functions. Results presented here suggest that patchouli alcohol possesses anti-influenza A (H2N2) virus properties, and therefore is a potential source of anti-influenza agents for the pharmaceutical industry.

## 1. Introduction

The influenza virus, which is one of the main causes of acute respiratory infections in humans, can lead to annual epidemics and infrequent pandemics. The two influenza pandemics of the 20^th^ century, “Asian Influenza (1957/H2N2)” and “Hong Kong Influenza (1968/H3N2)” resulted in the deaths of an estimated 2–3 million people globally [1,2]. Today, their descendants continue to cause the majority of influenza infections in humans [3]. So far as it is learned that the most effective antiviral drug is the neuraminidase (NA) inhibitor, which target the NA glycoproteins of influenza A and B virus [4,5].

The release of new virions from the infected cell is a key step in the influenza life cycle and need neuraminidase (NA) to cleave the α-ketosidic linkage between terminal sialic acid and an adjacent sugar residue [6]. The NA inhibitors were designed to prevent the key step by blocking the active site of enzyme and thus allow sufficient time for the host immune systems to remove infected viruses [7]. Consistent efforts have been devoted to the development of NA inhibitors, using the crystal structure of the N2 sub-type NA protein [8,9,10,11,12,13,14,15]. Indeed, oseltamivir (Tamiflu) is the representative NA inhibitor that has proven to be uniquely applicable oral drug in clinical practice for the treatment of influenza infection [4,8,9]. However, with an increase in medical use, the oseltamivir-resistant strains have been found and probably lead to a large scale outbreak of novel pandemic flu [16,17].

**Figure 1:**
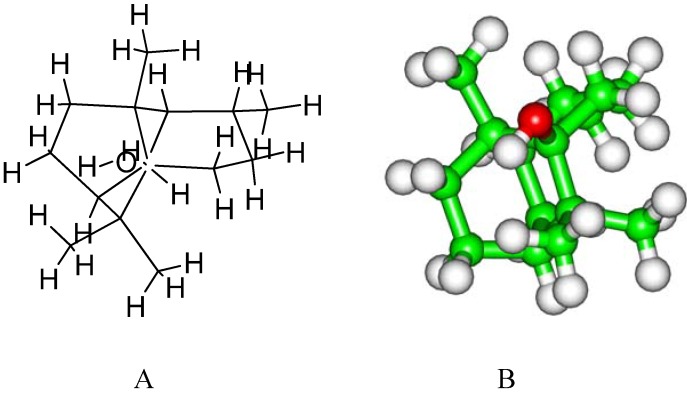
Chemical (**A**) and stereo (**B**) structures of patchouli alcohol.

Patchouli alcohol (Figure 1) has been well known for over a century. It is a major constituent of the pungent oil from the East Indian shrub *Pogostemon cablin (Blanco)* Benth, and widely used in fragrances. Patchouli oil is an important essential oil in the perfume industry, used to give a base and lasting character to a fragrance [16,17]. The essential oil is very appreciated for its characteristic pleasant and long lasting woody, earthy, and camphoraceous odor, as well as for its fixative properties, being suitable for use in soaps and cosmetic products [16,17]. The aerial part of *Pogostemon cablin* has wildly been used for the treatment of the common cold and as an antifungal agent in China [16,17]. Moreover, the plant is widely used in Traditional Chinese Medicine as it presents various types of pharmacological activity according to the composition of the oil [16,17]. Patchouli alcohol, as the major volatile constituent of patchouli oil, has been found to strongly inhibit H1N1 replication and weakly inhibit B/Ibaraki/2/85 replication [18]. 

To the best of our knowledge, the anti-influenza virus (H2N2) activities of patchouli alcohol have not been evaluated yet. Therefore, the aim of the present study was to evaluate the anti-influenza A virus (H2N2) activity of patchouli alcohol by MTT assay and mouse influenza model. On such basis, explicitly solvated docking and molecular dynamic (MD) methods were applied to investigative the binding mode involving patchouli alcohol with influenza virus NA protein. We anticipate that the insight into the understanding of inhibiting mechanism will be of value in the rational design of novel anti-influenza drugs.

## 2. Results and Discussion

### 2.1. Effect of patchouli alcohol against influenza A (H2N2) virus by MTT assay in vitro

First the efficacy of patchouli alcohol on influenza A (H2N2) virus replication and cell viability were examined. CC_50_ was used to express the cytotoxicity of patchouli alcohol on MDCK. The CC_50_ of patchouli alcohol was above 20 mM, which indicated that patchouli alcohol did not affect the growth of MDCK (Table 1). Thus, it seems that the antiviral effects of patchouli alcohol were not due to the cytotoxicity. 

**Table 1:** Anti-influenza A (H2N2) *virus* activity of patchouli alcohol compared with oseltamivir.

Compound	CC_50_ *^a^* (µM)	Influenza A (A/Udorn/307/72)	
		IC_50_^*b*^ (µM)	SI *^c^*
Patchouli alcohol	>20.0	4.03 ± 0.23 *	>4.96
Oseltamivir	>20.0	0.031 ± 0.012	>645.16

Values in this table represent the mean values (±SD) of three independent experiments (*P* < 0.01). ^a^ Cytotoxic effect was determined by MTT assay. CC_50_ was the concentration that showed 50% cytotoxic effects in MDCK cells; ^b^ Antiviral activity was determined by MTT assay. IC_50_ was the concentration that inhibited 50% of Influenza A [(Leningrad/134/17/1957) (H2N2)] virus replication in MDCK; ^c^ The selective index (SI) was calculated as CC_50_/IC_50_. The symbol * indicate a very significant difference *p* < 0.01 with respect to positive control (oseltamivir).

Moreover, patchouli alcohol was found to inhibit influenza A (H2N2) virus with an IC_50_ of 4.03 ± 0.23 µM. Based on the IC_50_ and CC_50_ values, the selectivity index (SI) was calculated as >4.96. It is reported that a SI of 4 or more is appropriate for an antiviral agent [18], suggesting that patchouli alcohol can be judged to have anti-influenza A (H2N2) virus activity.

Until now, it has been found that patchouli alcohol showed dose-dependent anti-influenza virus (A/PR/8/34, H1N1) activity, with an IC_50_ value of 2.635 µM. Furthermore, it showed weak activity against B/Ibaraki/2/85 (IC_50_ = 40.82 µM) [19]. With the addition of the above H2N2 inhibitory activity, we have a comprehensively view of the anti-influenza activity of patchouli alcohol.

### 2.2. Mode of anti-influenza A (H2N2) activity

Cells were pretreated with patchouli alcohol prior to virus infection (pretreatment cells), viruses were pretreated prior to infection (pretreatment virus), and patchouli alcohol was added during the adsorption period (adsorption) or after penetration of the viruses into cells (replication). Experiments were repeated independently three times and data presented are the average of three experiments. The symbols * indicated very significant difference *p* < 0.01 with respect to other mode (pretreatment virus, adsorption and pretreatment cell).

**Figure 2:**
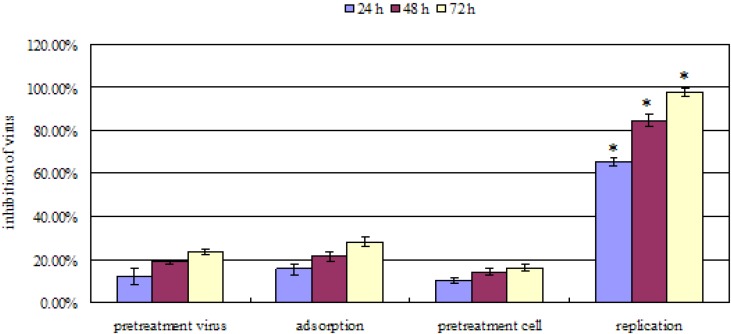
Antiviral effect of patchouli alcohol against influenza A (H2N2) virus by incubation at different periods of time during infection.

As shown in Figure 2, patchouli alcohol showed anti-influenza A (H2N2) virus activity in a time-dependent manner. It showed best antiviral activity when added at a concentration of 8 µM during the replication period with inhibition of the viral replication of 97.68% ± 2.09% for influenza A (H2N2) at 72 h. However, no significant effect was detected when patchouli alcohol was used for pretreatment of cells or viruses or when patchouli alcohol was only added during the adsorption phase. These results suggested that the inhibition of influenza A (H2N2) virus by patchouli alcohol appears to occur much more strongly after penetration of the virus into the cell. Besides, biochemical studies have indicated that the bioactivity of NA protein is essential determinant after the replication of influenza A (H2N2) virus [20,21,22]. Hence, we conclude that the function of NA protein may be suppressed by patchouli alcohol.

### 2.3. Anti-influenza A (H2N2) efficacy in mouse influenza model

To evaluate the toxicity of patchouli alcohol, the mean value of body weight of mice in each group was statistically analyzed. The mean weights of mice administered at the 2 mg/kg/dose oseltamivir, 2 mg/kg/dose patchouli alcohol and 10 mg/kg/dose of patchouli alcohol one time daily for 7 days were not significantly different compared with the normal control mice, showing no toxicity of patchouli alcohol and oseltamivir within the testing concentration (*P* > 0.05). Physiological status was observed in virus infection mice. Three days after viral infection, some mice, especially mice in the H2N2 infected control group showed changes in behavior, such as a tendency to huddle, diminished vitality, and ruffled fur, *etc.* In the mouse influenza model, viral infection leads to loss of body weight and high mortality. Therefore, the efficacy of patchouli alcohol and oseltamivir were evaluated on the basis of survival rate measured for 15 days post-infection, for treated infected animals relative to untreated infected (control) animals. A comparison of efficacy of patchouli alcohol and oseltamivir *in vivo* mouse influenza model (oral treatment) showed that at a dose of 5 mg/kg/day, patchouli alcohol showed obvious protection against the influenza virus, as the mean day to death was detected as 11.8 ± 1.1 (Table 2). When the dose was lowered to 1 mg/kg/day, patchouli alcohol showed weaker protection (measured by Survivors/total) than that of 5 mg/kg/day, the mean day to death was 7.5 ± 1.8. Whereas oseltamivir at this dose level (1 mg/kg/day) showed 50% protection (measured by survivors/total) against the influenza virus. In the H2N2 infected control group, there were no survivors. In view of both *in vitro* and *in vivo* data, we conclude that patchouli alcohol could be used in the treatment of human influenza virus infections.

**Table 2:** Comparison of efficacy of patchouli alcohol with oseltamivir in the *in vivo* mouse influenza model (oral treatment).

Group	Dose, mg/kg/day (q.d.)	Survivors/total	Mean day to death
Patchouli alcohol	5	7/10 *	11.8 ± 1.1 *
	1	2/10 *	7.5 ± 1.8 *
Oseltamivir	1	5/10*	9.1 ± 0.3 *
Control	-	10/10	-
H2N2 infected control	-	0/10	5.2 ± 1.6

* *p* < 0.001 *versus* H2N2 infected control; ^a^ Mean day to death of mice dying prior to day 15.

### 2.4. In silico inhibition mechanism of patchouli alcohol

**Figure 3:**
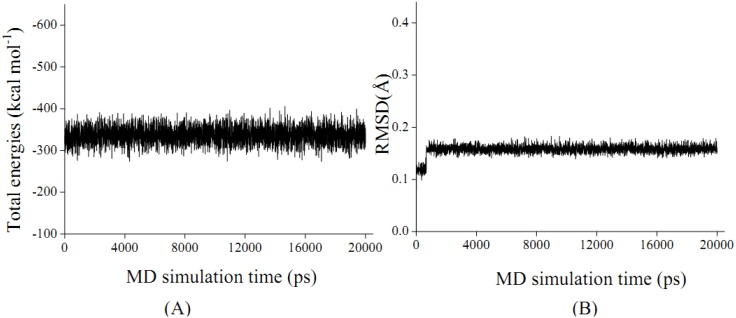
The total energies of ensemble (Total energies, A) and time-evolution backbone-atom root mean square deviations (RMSD, B) during the molecular dynamic (MD) simulations for the NA protein complexed with patchouli alcohol.

Based on the above experiment data, patchouli alcohol is determined to be bound within NA protein. As the total energies and backbone root-mean-square-deviations (RMSD) in Figure 3 indicate, the energy-minimized patchouli alcohol-NA complex has been in equilibrium since about 0.5 ns, and then retains quite stable in the last 19.5 ns. It is consistent with the previous MD results of other NA inhibitors [23,24,25,26,27,28]. Accordingly, the geometric and energetic analyses were made on the average structures of 0.5~20.0 ns MD trajectories, where the system has been already at equilibrium. 

**Figure 4:**
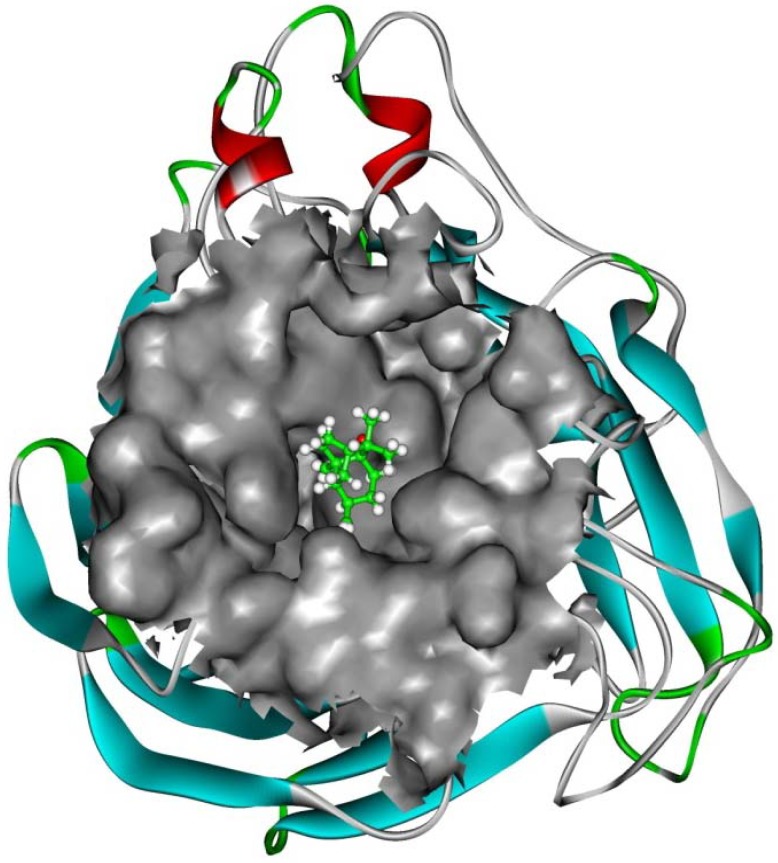
Patchouli alcohol bound at the NA active site. The Connolly surfaces of the NA active site (in grey) are created using the InsightII 2005 scripts. The patchouli alcohol is represented by ball and stick model. Ribbon colors: Helices, beta sheets, turns and random coils are in red, cyan, green and white, respectively.

**Figure 5:**
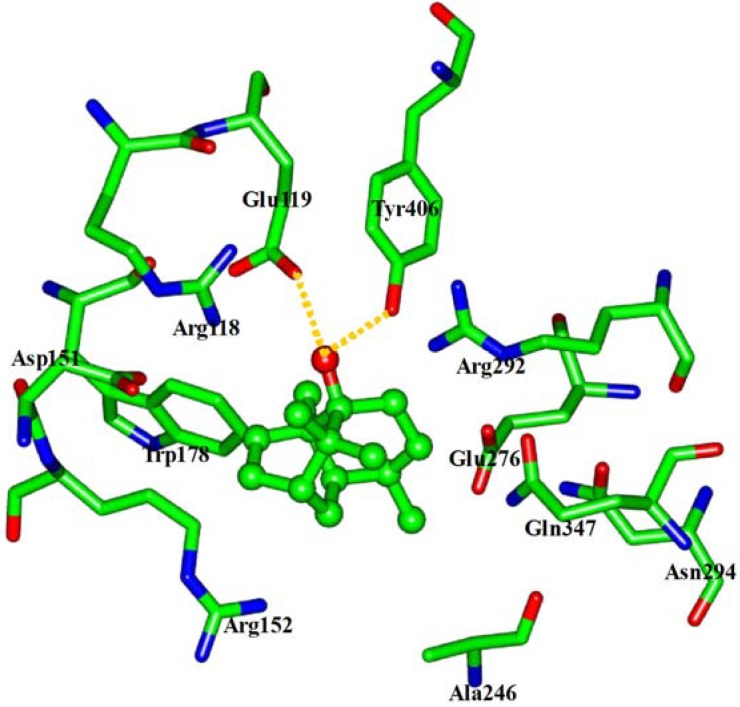
View of the binding mode of patchouli alcohol with the NA active-site residues. Key residues are represented by stick models. Patchouli alcohol is represented by ball and stick model. The hydrogens were avoided for readability. The important H-bonds are labeled in the dashed gold lines.

**Figure 6:**
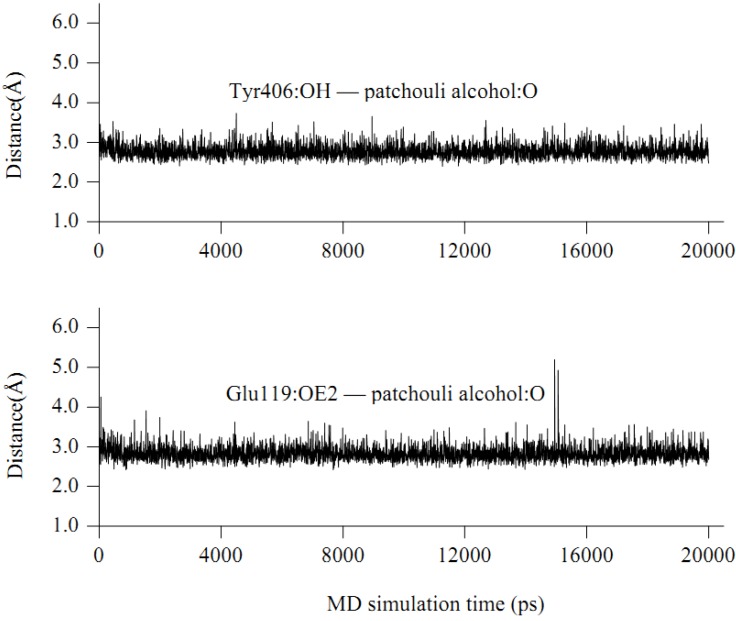
Some time-evolution distances between patchouli alcohol and NA residues.

The interaction energy (E*_inter_*) of patchouli alcohol with NA was calculated at −40.38 kcal mol^−1^, where the vdW rather than electrostatic interactions were found to play a dominant role, contribute to about 72% (−29.18 kcal mol^−1^). As shown in Figure 4, the patchouli alcohol was bound at the active site which also bound to oseltamivir and zanamivir [28]. As Figure 5 shows, the oxygen atom of patchouli alcohol was oriented towards the sidechains of residues Glu119 and Tyr406, with one H-bond formed with each residue. The values of distances in Figure 6 further reveal that the docked complex remains rather stable throughout the simulation, with the average distances of Glu119:OE2 — patchouli alcohol:O and Tyr406:OH — patchouli alcohol:O less than 2.8 Å. The sum contributions (E*_sum_*) of residues Glu119 and Tyr406 amounted to −8.46 and −7.37 kcal mol^−1^, respectively (Table 3). Besides, patchouli alcohol was stabilized by residues Arg118, Asp151, Arg152, Trp178, Ala246, Glu276, Arg292, Asn294 and Gln347, especially residues Asp151, Arg152 and Glu276 (Figure 5 and Table 3). As a matter of fact, residues Asp151, Arg152, Glu119, Glu276 and Tyr406 of the NA protein have already received enough attention from rational drug designs [14,30,31]. The catalytic residues Asp151, Arg152 and Glu276 are crucial to the NA functions and the residues Glu119 and Tyr406 are important to stabilize the NA active sites [32,33]. It suggests that the NA functions will be affected by the presence of patchouli alcohol, consistent with the above experiments. Patchouli alcohol matches with the NA active site and has an acceptable interaction energy. Considering the obvious structure discrepancies against current NA inhibitors, it represents an ideal lead compound for the designs of novel anti-influenza agents.

**Table 3:** The vdW, electrostatic and sum interaction energies (E*_vdW_*, E*_ele_* and E*_sum_*) between patchouli alcohol and the key active-site residues of NA protein *^a^.*

Residue	E *_vdW_*	E *_ele_*	E *_sum_*
Arg118	−2.03	0.63	−1.40
Glu119	−2.18	−6.28	−8.46
Asp151	−2.08	−0.53	−2.61
Arg152	−3.01	0.46	−2.55
Trp178	−1.83	0.01	−1.82
Ala246	−1.47	0.12	−1.35
Glu276	−2.04	−0.28	−2.32
Arg292	−3.51	1.10	−2.41
Asn294	−0.92	0.16	−0.76
Gln347	−1.91	0.07	−1.84
Tyr406	−3.07	−4.30	−7.37

*^a^* Energy units in kcal mol^−1^.

## 3. Experimental

### 3.1. Materials

Patchouli alcohol and oseltamivir were obtained from Sigma Chemical Co. (St. Louis, MO, USA, purity > 99%) and was stored in glass vials with Teflon sealed caps at −20 ± 0.5 °C in the absence of light.

### 3.2. Cell cultures

MDCK (Madin-Darby canine kidney) was purchased from Harbin Veterinary Research Institute (Harbin, Heilongjiang, China). The cells were grown in monolayer culture with Eagle’s minimum essential medium (EMEM) supplemented with 10% fetal calf serum (FCS), 100 U/mL penicillin and 100 μg/mL streptomycin. The monolayers were removed from their plastic surfaces and serially passaged whenever they became confluent. Cells were plated out onto 96-well culture plates for cytotoxicity and anti-influenza assays, and propagated at 37 °C in an atmosphere of 5% CO_2_.

### 3.3. Viruses

The influenza strain A/Leningrad/134/17/1957 H2N2) was purchased from National Control Institute of Veterinary Bioproducts and Pharmaceuticals (Beijing, China). Virus was routinely grown on MDCK cells. The stock cultures were prepared from supernatants of infected cells and stored at −80 °C.

### 3.4. Cytotoxicity assay

The cellular toxicity of patchouli alcohol on MDCK cells was assessed by the MTT method. Briefly, cells were seeded on a microtiter plate in the absence or presence of various concentrations (20 µM – 0.0098 µM) of patchouli alcohol (eight replicates) and incubated at 37 °C in a humidified atmosphere of 5% CO_2_ for 72 h. The supernatants were discarded, washed with PBS twice and MTT reagent (5 mg/mL in PBS) was added to each well. After incubation at 37 °C for 4 h, the supernatants were removed, then 200 μL DMSO was added and incubated at 37 °C for another 30 min. After that the plates were read on an ELISA reader (Thermo Molecular Devices Co., Union City, USA) at 570/630 nm. The mean OD of the cell control wells was assigned a value of 100%. The maximal non-toxic concentration (TD_0_) and 50% cytotoxic concentration (CC_50_) were calculated by linear regression analysis of the dose-response curves generated from the data.

### 3.5. Anti-influenza A (H2N2) activity in vitro

Inhibition of virus replication was measured by the MTT method. Serial dilution of the treated virus was adsorbed to the cells for 1 h at 37 °C. The residual inoculum was discared and infected cells were added with EMEM containing 2% FCS. Each assay was performed in eight replicates. After incubation for 72 h at 37 °C, the cultures were measured by MTT method as described above. The concentration of patchouli alcohol and oseltamivir which inhibited virus numbers by 50% (IC_50_) was determined from dose-response curves.

### 3.6. Mode of anti-influenza A (H2N2) activity

Cells and viruses were incubated with patchouli alcohol at different stages during the viral infection cycle in order to determine the mode of antiviral action. Cells were pretreated with patchouli alcohol before viral infection, viruses were incubated with patchouli alcohol before infection and cells and viruses were incubated together with patchouli alcohol during adsorption or after penetration of the virus into the host cells. Patchouli alcohol was always used at the nontoxic concentration. Cell monolayers were pretreated with patchouli alcohol prior to inoculation with virus by adding patchouli alcohol to the culture medium and incubation for 1 h at 37 °C. The compound was aspirated and cells were washed immediately before the influenza A (H2N2) inoculum was added. For pretreatment virus, Influenza A (H2N2) was incubated in medium containing patchouli alcohol for 1h at room temperature prior to infection of MDCK cells. For analyzing the anti-influenza A (H2N2) inhibition during the adsorption period, the same amount of influenza A (H2N2) was mixed with the drug and added to the cells immediately. After 1 h of adsorption at 37 °C, the inoculum was removed and DMEM supplemented with 2 % FCS were added to the cells. The effect of patchouli alcohol against influenza A (H2N2) was also tested during the replication period by adding it after adsorption, as typical performed in anti-influenza A (H2N2) susceptibility studies. Each assay was run in eight replicates. 

### 3.7. Anti-influenza A (H2N2) efficacy in mouse influenza model

Kunming mice, weighing 18–22 g (6 weeks of age) were purchased from Harbin Veterinary Research Institute Animal Co., Ltd. (Harbin, Heilongjiang, China). First, the toxicity of patchouli alcohol and oseltamivir was assessed in the healthy mice by the loss of body weight compared with the control group (2% DMSO in physiological saline). The mice were orally administered with 10 mg/kg/dose patchouli alcohol, 2 mg/kg/dose patchouli alcohol or 2 mg/kg/dose oseltamivir (dissolved in 2% DMSO in physiological saline) one time daily for 7 days. The weight of mice was determined daily. We conducted procedures according to Principle of Laboratory Animal Care (NIH Publication No. 85 – 23, revised 1985) and the guidelines of the Peking University Animal Research Committee. 

Kunming mice were anesthetized with isoflurane and exposed to virus (A/Leningrad/134/17/1957) by intranasal instillation. Drugs were prepared in 2% DMSO in physiological saline and administered 4 h prior to virus exposure and continued daily for 5 days. All mice were observed daily for changes in weight and for any deaths. Parameters for evaluation of antiviral activity included weight loss, reduction in mortality and/or increase in mean day to death (MDD) determined through 15 days.

### 3.8. Computational methods

The N2 sub-type neuraminidase crystal structure (PDB code 1IVD) was obtained from the RCSB Protein Data Bank [34]. For convenience, the structure is named as NA hereafter. Geometry and partial atomic charges of the patchouli alcohol (Figure 1) were calculated with the Discover 3.0 module (Insight II 2005) [35] by applying the BFGS algorithm [36] and the consistent-valence force-field (CVFF), with a convergence criterion of 0.01 kcal mol^−1^ Å^−1^. The docking and molecular dynamics (MD) simulations were performed by the general protocols in the Insight II 2005 software packages, consistent with the previous literatures [24,26,28,35,37,38,39]. During the MD simulations, the canonical ensemble (NVT) was employed at normal temperature (300 K). The MD temperature was controlled by the velocity scaling thermostat [40]. Integrations of the classical equations of motion were achieved using the Verlet algorithm. The systems were solvated in a large sphere of TIP3P water molecules [40] with the radius of 35.0 Å, which is enough to hold the ensembles [40]. The MD trajectories were generated using a 1.0-fs time step for a total of 20.0 ns, saved at 5.0-ps intervals. The interaction energies of patchouli alcohol with NA and the respective residues at the NA active site were calculated by the Docking module [35], over the 0.5~20.0 ns MD trajectories. 

### 3.9. Statistical analysis

All results are expressed as mean values ± standard deviations (SDs) (n = 3). The significance of difference was calculated by one-way analysis of variance, and values *p* < 0.001 were considered to be significant.

## 4. Conclusions

In conclusion, patchouli alcohol possesses anti-influenza A (H2N2) virus activity via interference with the NA function that cleaves the α-glycosidic bond between sialic acid and glycoconjugate. Our results provide the promising information for the potential use of patchouli alcohol in the treatment of influenza A (H2N2) virus infectious disease. Further mechanistic studies on the anti-influenza A virus activity are needed to support this point of view. 
